# The Role of Prep1 in the Regulation of Mesenchymal Stromal Cells

**DOI:** 10.3390/ijms20153639

**Published:** 2019-07-25

**Authors:** Giorgia Maroni, Daniele Panetta, Raffaele Luongo, Indira Krishnan, Federica La Rosa, Daniela Campani, Piero Salvadori, Patricia Iozzo, Francesco Blasi, Dmitry Penkov, Elena Levantini, Maria Cristina Magli

**Affiliations:** 1 Institute of Biomedical Technologies, National Research Council (CNR), 56124 Pisa, Italy; 2 Cancer Science Institute, National University of Singapore, Singapore 117599, Singapore; 3 Institute of Clinical Physiology, National Research Council (CNR), 56124 Pisa, Italy; 4 Department of Medicine, Harvard Medical School, Harvard University, Boston, MA 02215, USA; 5 Pathology Unit, University of Pisa, 56024 Pisa, Italy; 6 The FIRC Institute of Molecular Oncology (IFOM), 20139 Milano, Italy; 7 Institute of Experimental Cardiology, National Medical Research Center for Cardiology, 121552 Moscow, Russia; 8 Hematology/Oncology, Beth Israel Deaconess Medical Center, Boston, MA 02215, USA; 9 Harvard Stem Cell Institute, Harvard University, Boston, MA 02215, USA

**Keywords:** mesenchymal stromal cells (MSC), adipogenesis, osteogenesis, homeobox gene, murine models, single cell RNA sequencing, in vivo imaging

## Abstract

Molecular mechanisms governing cell fate decision events in bone marrow mesenchymal stromal cells (MSC) are still poorly understood. Herein, we investigated the homeobox gene *Prep1* as a candidate regulatory molecule, by adopting *Prep1* hypomorphic mice as a model to investigate the effects of *Prep1* downregulation, using in vitro and in vivo assays, including the innovative single cell RNA sequencing technology. Taken together, our findings indicate that low levels of *Prep1* are associated to enhanced adipogenesis and a concomitant reduced osteogenesis in the bone marrow, suggesting Prep1 as a potential regulator of the adipo-osteogenic differentiation of mesenchymal stromal cells. Furthermore, our data suggest that in vivo decreased *Prep1* gene dosage favors a pro-adipogenic phenotype and induces a “browning” effect in all fat tissues.

## 1. Introduction

Prep1 is a key developmental regulator, as its complete in vivo inactivation is embryonic lethal at the epiblast stage [1]. However, hypomorphic *Prep1^i/i^* embryos, that express ~2% of normal *Prep1* mRNA, have a milder phenotype, with frequent embryonic lethality at E17.5 [2]. The few *Prep1^i/i^* mice that reach adulthood exhibit impairment in T and B cells differentiation, although the mechanisms are still unclear [3], and develop B- and T- cell tumors [4]. Foetal liver *Prep1^i/i^* hematopoietic stem cells (HSCs) are rapidly exhausted, but still able to inefficiently repopulate irradiated hosts [5]. However, no phenotype is observed in mice in which *Prep1* null deletion is studied in adult HSCs [6,7], hinting that also mesenchymal stromal cells may contribute to hematopoietic phenotypes. Therefore, we studied bone marrow (BM) mesenchymal stromal cells in *Prep1^i/i^* mice, where the level of *Prep1* is drastically reduced.

Mesenchymal stem cells (MSCs), also called mesenchymal stromal cells, have attracted great interest for their biological properties as potentially powerful tools in the field of regenerative medicine [8,9,10]. MSCs are multipotent clonogenic cells that can give rise, both in vivo and in vitro, to cells of different mesenchymal tissues such as bone, cartilage and fat [11]. Furthermore, MSCs and their differentiated progeny, in particular osteoblasts and adipocytes, are components of the bone marrow niche, in which the HSCs reside, and contribute to regulating their function [12,13,14].

We previously provided the first evidence that the transcriptional regulator Prep1 may play a role in the differentiation of murine MSCs. Indeed, we have recently shown that *Prep1* downregulation in both ex vivo bone marrow-derived MSCs and in the pre-adipocytic cell line 3T3-L1 significantly increases their adipogenic differentiation ability [15]. Interestingly, undifferentiated *Prep1^i/i^* MSCs showed higher gene expression levels of adipogenic markers, as compared to *Prep1^+/+^* control cells. Furthermore, following adipogenic induction, *Prep1^i/i^* MSCs differentiated much faster than wild type (wt) MSCs. These observations suggest that *Prep1* downregulation itself favours commitment of MSCs towards adipogenic fate, implying that Prep1 normally acts as an inhibitor for the adipogenic differentiation program.

In order to better understand the role of Prep1 in the regulation of mesenchymal/stromal tissues we have herein further investigated the effects of *Prep1* downregulation using in vitro assays, in vivo imaging techniques, and the innovative single cell RNA sequencing (scRNAseq) technology, performed on freshly isolated cells.

Our results show that downregulation of *Prep1* affects both the adipogenic and the osteogenic cell compartments. Histological analysis of bone marrow cells provide further evidence that reduced levels of *Prep1* induce an increase in the percentage of fat cells. Importantly, scRNAseq analysis provides initial evidence that *Prep1^i/i^* BM cells display defective osteogenesis, as assessed by the great reduction of a specific transcriptional cluster/subpopulation, identified mainly in wt BM. Accordingly, in vitro cultured *Prep1^i/i^* MSCs show decreased ability to generate mature osteoblasts, upon osteogenic induction. Moreover, our data show that *Prep1* downregulation induces alterations also in in vivo fat depots, such as decreased size in white and brown adipocytes, and a higher brown adipose tissue (BAT) radiodensity, as assessed by micro-CT analysis, which might contribute to explain its favourable metabolic action, as recently revised in Oriente et al. [16].

Taken together, our findings indicate that Prep1 is involved in the regulation of mesenchymal/ stromal tissues, playing an important role in adipogenesis and provide initial evidence that it may be involved in the osteogenic process as well. Since it is widely accepted that adipogenic and osteogenic differentiation are mutually exclusive processes, we can speculate that Prep1 may act at the level of the adipo-osteogenic switch.

## 2. Results

To confirm our previous data on cultured MSCs, hinting to a role for *Prep1* in adipogenesis, we have further analysed mice carrying the Prep1^i/i^ mutation, and compared them to their wt siblings.

### 2.1. Histologic Analysis of BM and Fat Tissues

**Figure 1:**
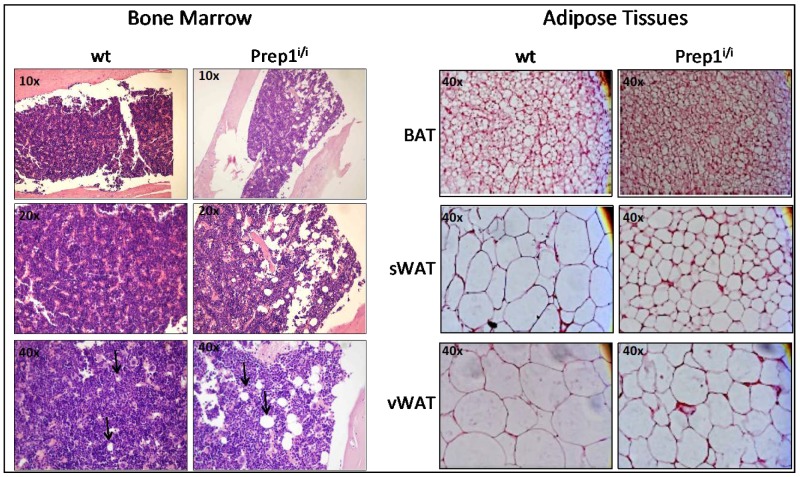
Hematoxylin Eosin staining shows differences in adipose depots between wt and Prep1^i/i^ mice. HE staining has been performed on (**A**) bone marrow sections of wt (left panel) and Prep1^i/i^ mice (right panel), 10× (upper panel), 20× (middle panel) and 40× (lower panel) magnifications are shown. Black raws indicate the presence of adipocytes. HE on (**B**) different adipose tissues, i.e. Brown Adipose Tissue (BAT) (upper panel), subcutaneous White Adipose Tissue (sWAT) (middle panel) and visceral White Adipose Tissue (vWAT) (lower panel), 40× magnification is shown.

As a first step, we performed histological analysis of freshly explanted bone marrows (femurs), as well as brown interscapular (BAT) and white adipose tissues (subcutaneous sWAT, and visceral vWAT). Figure 1 (left panels) shows that wt and hypomorphic BM display a rather different cellular composition, in that wt BM appears more compact, with a dense cellularity, as compared to its hypomorphic counterpart. *Prep1^i/i^* BM is indeed characterized by a substantial presence of large adipocytes, as shown at 40× magnification, which are most probably responsible for the looser morphology observed in the hypomorphic BM.

Histological sections of BAT and WAT (subcutaneous and visceral) derived from normal and mutant mice (Figure 1, right panels) show that, in all three fat tissues of *Prep1^i/i^* mice, adipocytes appear more numerous but smaller in size.

### 2.2. Whole Body Analysis by Micro-CT Imaging Techniques of WT and Prep1^i/i^ Mice

**Figure 2:**
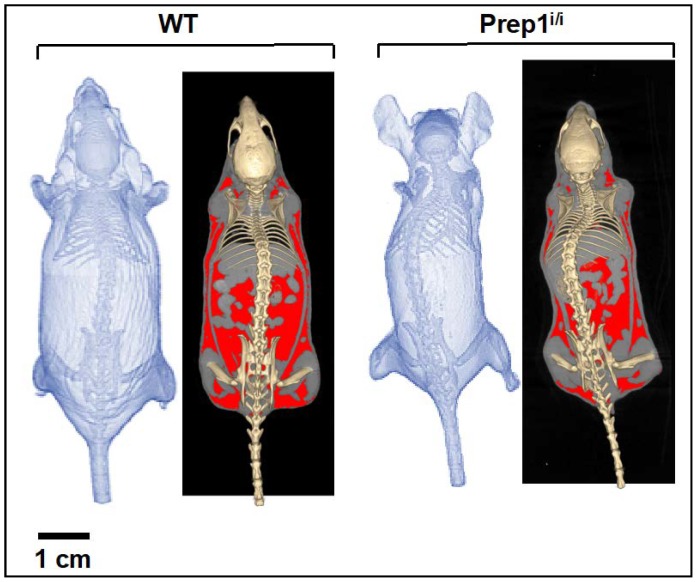
Volumetric rendering of the whole body micro-CT reconstructions indicates on average a smaller, lighter, shorter and less adipose tissue in hypomorphic mice. A wt mouse (left) and a Prep1^i/i^ mouse (right) are represented. In the right panel of each group, the highlighted region represents the segmented adipose tissue.

Therefore, we proceeded to compare the entire body-wide distribution of fat depots by in vivo whole-body analysis of wt vs. *Prep1^i/i^* mice, adopting micro-CT imaging techniques. Macroscopic morphological differences are observed between the hypomorphic and wild type groups, as exemplified in Figure 2 and detailed in Table 1. In particular, the weight and total volume of the *Prep1^i/i^* mice are on average 15% (*p* = 0.029) and 16% lower (*p* = 0.026) than their control littermates, respectively. This is mainly due to the significant difference in total fat volume, which is 39% lower in *Prep1^i/i^* vs. wt (*p* = 0.029). No significant differences are instead observed in volume and density of the lean mass component.

**Figure 3:**
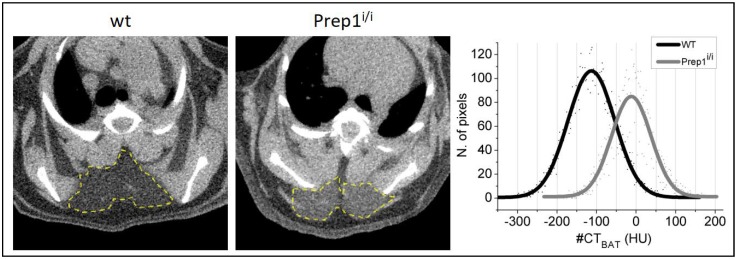
Hypomorph subjects show a higher density of the interscapular BAT than wt. Transaxial slices of the micro-CT scans. Figure shows density of interscapular BAT of the wt mouse (left) and the Prep1^i/i^ mouse (center). The histogram on the right panel shows the distribution of gray level intensities inside the highlighted BAT Region of Interest (ROI).

In contrast, important differences are found in interscapular BAT radiodensity (Table 1 and Figure 3) i.e., −132 ± 36 Hounsfield units (HU) for *Prep1^i/i^* vs. −180 ± 46 HU for wt (*p* = 0.013), indicative of lower lipid accumulation in BAT of *Prep1^i/i^* vs. wt mice [17]. Interscapular BAT volume was instead similar between groups.

### 2.3. Single Cell Transcriptomics of BM Mesenchymal Stromal Cells

Mesenchymal stromal cells are a highly heterogeneous cell population, for which there are a lack of surface and molecular markers that identify specific stromal stem/progenitor subpopulations. Therefore, to characterize differences in the MSC populations of wt and *Prep1* hypomorphic mice, we used state-of-the-art single cell transcriptomics analysis, a useful resource to identify unique cellular differentiation states/clusters characterized by defined transcriptional states.

**Figure 4:**
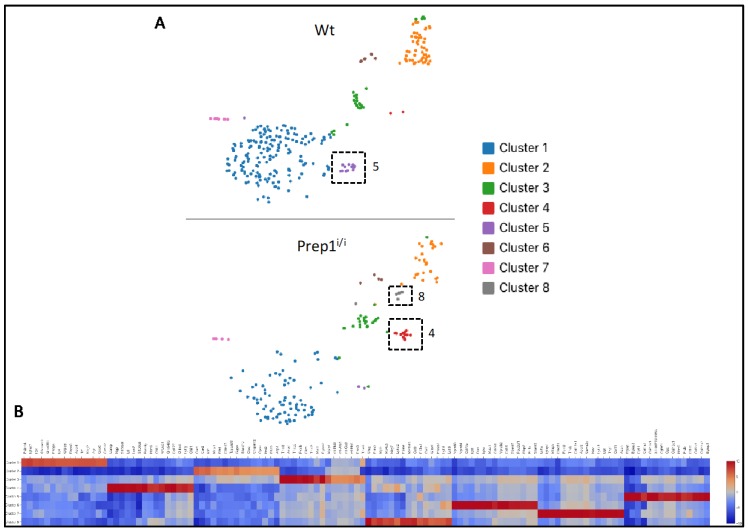
Single Cell RNA Sequencing reveals different BM cell composition between wt and hypomorphic samples. (**A**) t-SNE plot of single Cell RNA sequencing, performed on wt (upper panel) and Prep1^i/i^ (lower panel) fresh Bone Marrow Stromal Cells, shows eight distinct transcriptional clusters, through Loupe Cell Browser visualization. Clusters specific for each sample are highlighted by dotted squares. (**B**) The heatmap shows unique molecular signatures displayed by each cluster.

The *Prep1^i/i^* mice are rare survivors of heterozygous crosses since 75% of the homozygous embryos die at E17.5 [2]. We used BM cells from one *Prep1^i/i^* mouse and its wt sibling, together with dissociated/digested bone chips and we depleted the haematopoietic and endothelial cell components by FACS. CD45^−^/CD31^−^/Ter119^−^ cells were subsequently subjected to analysis using 10X Genomics microfluidic systems. cDNA libraries from RNA obtained from the two samples were sequenced and the raw sequencing data processed, using the Cell Ranger bioinformatics pipeline, and visualized by Loupe Cell browser. The sequence reads from the two mice were pooled to build a unique database which amounted to a total of 363,478 reads from 454 cells, with a median number of 2017 genes per cell. Deconvolution analysis was consistent with a total of eight transcriptional clusters/subpopulations (Figure 4A), of which one is mainly present in wt (cluster #5), one mostly detected in *Prep1^i/i^* (cluster #4) and one exclusively observed in *Prep1^i/i^* BM (cluster #8). The heatmap of the gene expression profile of the eight clusters (Figure 4B) shows that each cluster is characterized by a unique transcriptional signature and, therefore, represents specific subpopulations.

**Figure 5:**
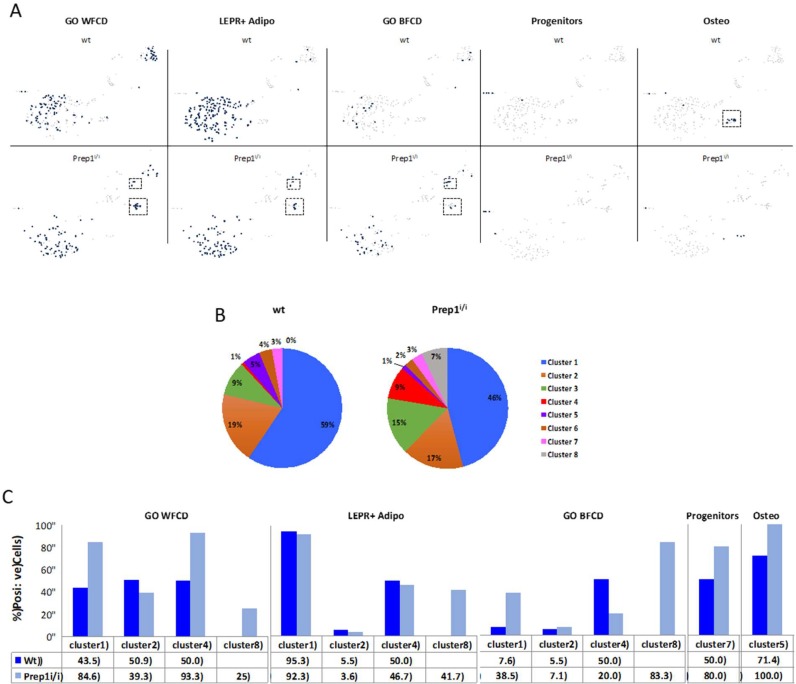
Cluster annotation highlights adipogenic and osteogenic subpopulations. (**A**) Annotation, using specific gene lists (GO White Fat Cell Differentiation, LEPR+ Adipo, GO Brown Fat Cell Differentiation, Progenitors and Osteo), shows the presence of specific cell types in each cluster. Dotted squares highlights clusters (most uniquely expressed per each genotype). (**B**) Pie charts depict percentages of cells present in each cluster. (**C**) Histograms show the percentage of cells per cluster which are positive to the indicated annotation. Blue bars indicate wt cells, light blue bars refer to hypomorphic cells. GO, Gene Ontology; WFCD, White Fat Cell Differentiation; LEPR, Leptin Receptor; BFCD, Brown Fat Cell Differentiation.

To shed some light on the cellular identity of the clusters, we evaluated the expression and/or co-expression of different signatures known to be associated with adipogenic, osteogenic or stem/early progenitor cells. First, we annotated our data set by using genes belonging to the Gene Ontology (GO) White Fat Cell Differentiation (WFCD) category (Table 2). As shown in the t-SNE plot (Figure 5A) and histograms (Figure 5C), both wt and *Prep1^i/i^* clusters #1 and #2 appear positive for WFCD, as well as clusters #4, and the unique *Prep1^i/i^* #8. Percentages of cells present in these clusters are indicated in the pie charts depicted in Figure 5B.

We also used the combination of Leptin Receptor (LEPR) co-expression with that of a selected group of adipogenesis-associated genes (Table 2), recently exploited by Tikhonova et al. [18] to analyse single cell transcriptomics of BM cells (LEPR^+^ Adipo signature). LEPR^+^ Adipo cells are mostly present (Figure 5A,C) in clusters #1 in both wt and *Prep1^i/i^* BM; at low frequency in clusters #2; in clusters #4; and in the hypomorphic cluster #8.

A similar analysis, using the Brown Fat Cell Differentiation (BFCD) GO category (Table 2), showed positive cells within cluster #1, cluster #2 and cluster #4 of both wt and *Prep1^i/i^* BM (Figure 5A,C). In addition, they were expressed in *Prep1^i/i^* cluster #8.

Overall, our data highlight that genes associated with the adipogenic lineage are mostly identified in clusters #1, while, to a lesser extent, in cluster #2, #4, and the *Prep1^i/i^* specific #8.

Figure 5 also depicts that cells present in cluster #7 of both wt and mutant BM display a ≥50% co-expression of Platelet Derived Growth Factor Receptor alpha (PDGFRα) and Stem Cell Antigen 1 (Sca1), markers of mesenchymal stem/early progenitor cells [19].

Finally, using a combination of osteogenic genes (Table 2) we identified cluster #5 as enriched for cells of osteogenic nature. Indeed, cluster #5 is more highly represented in wt (5%) versus the hypomorphic (1%) BM (Figure 5B), suggesting that *Prep1* down regulation may affect also some mesenchymal osteogenic-prone subpopulation.

For some subpopulations (clusters #3 and #6) no gene signature was expressed with sufficient specificity to directly allow any defined lineage association/inference.

In our annotated clusters, *Prep1* was expressed in wt clusters #1, #5 and mainly in #2, whereas it was virtually undetectable in *Prep1^i/i^* BM, as expected [2] (Supplementary Figure S1). Therefore, although *Prep1* is considered a ubiquitously expressed gene [20] its expression is highly variable in individual cells of the mouse BM.

### 2.4. Comparative In Vitro Studies on Osteogenic Differentiation of wt and Prep1^i/i^ MSCs

**Figure 6:**
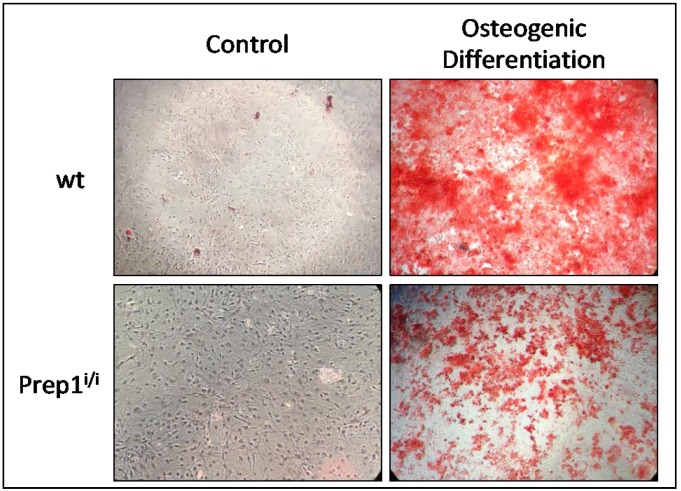
Alizarin Red O’Staining highlights altered osteogenic ability in culture Prep1^i/i^ cells. Staining is used to evaluate in culture osteogenic differentiation efficiency. Analysis has been performed on undifferentiated wt (upper left) and Prep1^i/i^ (lower left) cells as negative controls and on cells at terminal osteogenic differentiation, wt (upper right) and Prep1^i/i^ (lower right). Red staining labels the extracellular matrix secreted by mature osteobalsts.

Since our scRNA data hint to an osteogenic population being majorly detectable in wt BMs, we next compared the ability of wt and *Prep1^i/i^* MSCs to differentiate along the osteogenic lineage. Figure 6 shows that hypomorphic MSCs exhibit lower osteogenic differentiation ability, as assessed by Alizarin red staining, in agreement with our single cell transcriptomics.

## 3. Discussion

In recent years we have investigated the role of the homeobox-containing transcription factor Prep1 in the control of BM development. We first discovered an important effect of Prep1 on the viability and proliferation of foetal hematopoietic stem cells [5]. Given the critical relevance of the cross-talk between hematopoietic and stromal cells, we have recently addressed the question of whether Prep1 might also be an important regulator in the mesenchymal stromal system [15]. First, we observed that *Prep1* is inversely expressed during in vitro differentiation of wt MSCs: it is downregulated following in vitro adipogenic induction [15] and upregulated after osteogenic stimuli (our unpublished results). Furthermore, as *Prep1* complete inactivation is early-embryonic lethal [1], our functional studies in adult hypomorphic *Prep1^i/i^* mutants [2] showed that *Prep1^i/i^* MSCs display faster and more efficient in vitro adipogenic ability after specific stimulation, indicating that hypomorphic MSCs are already poised towards fat cell differentiation [15]. As our in vitro studies pointed to Prep1 as an important regulator of adipogenesis, we have herein verified this hypothesis in the BM, as well as in BAT, and subcutaneous and visceral WAT.

Indeed our results show that *Prep1^i/i^* bone marrow mesenchymal stromal cells display alterations as compared to their wt counterpart: i) histological analysis shows that reduced *Prep1* is associated with an increased frequency of adipocytes *in vivo;* ii) single cell RNA sequencing reveals important differences in the landscape of BM stromal cells between wt and hypomorphic milieus iii) *Prep1^i/i^* MSCs, besides the previously shown increase in adipogenic potential [15], display a diminished capacity to differentiate in vitro towards the osteogenic lineage.

Moreover, *Prep1* deficiency affects the adipose tissues, decreasing the WAT volume and concomitantly increasing BAT density, as well as inducing changes in the cellular size of the fat depots.

In the present study a novel observation was the histological evidence of bone marrow adiposity, as characterized by adipocytes with large lipid depots, apparently disrupting the surrounding BM structure in hypomorphic mice. This result supports and complements the higher adipogenicity of hypomorphic BM cells we previously reported [15]. Our prior observation was obtained on cell lines and in vitro cultures of MSCs, which not always reflect the composition of in vivo mesenchymal compartments. Indeed, whether in vitro data reproduce in vivo characteristics is still a highly controversial issue. Therefore, our in vivo analyses provide the first evidence that changes in *Prep1* gene dosage are related to alterations in mesenchymal cell populations.

It is still unclear whether BM adipocytes are distinct from white, brown and beige adipocytes, representing a fourth class of adipocytes, or if they are lineage-related to cells of other fat depots [21]. Moreover, the process of BM adipogenesis is still poorly understood, and cell surface phenotype and gene signatures of BM adipocyte precursors are not available. Therefore, to compare non-hematopoietic BM components from wt and *Prep1* hypomorphic background we took advantage of the single cell RNA seq technology, which provides a screenshot into transcriptional landscapes of complex tissues, such as the mesenchymal system. Our data highlighted commonalities and differences in the bone marrow mesenchymal milieu of the two genotypes. Overall, deconvolution analysis identified a total of eight clusters, of which one (#8) is exclusively present in *Prep1^i/i^*.

In the following discussion, we do not imply that the clusters identified represent pure cell populations as they might still be further subdivided once additional cell markers become available.

To interrogate the adipogenic nature of the various clusters we used combined transcriptional profiles. In particular, we adopted not only the White Fat Cell Differentiation and Brown Fat Cell Differentiation categories from Gene Ontology, derived from body fat depots, but also recently available single cell RNAseq data sets from FACS purified normal BM cells (LEPR^+^ Adipo signature) [18]. Such analyses produced concordant results allowing for the identification of cluster #1 as highly enriched for the adipogenic signatures, in both genotypes (Figure 5). In addition, our data highlight the expression of adipogenic genes in clusters #4 and #8 as well. Noteworthy, in wt BM cluster #4 is almost undetectable, and cluster #8 is missing, indicating that these two subpopulations arise and/or expand in the absence of physiological *Prep1* levels. These data are consistent with our previous observation showing upregulation of the adipogenic differentiation program in *Prep1* downregulated cells.

Positivity of cluster #8 for BFCD category is mostly due to the high levels of expression of *Plac8*, which has been reported as key upstream molecule in the brown adipogenic regulatory network [22]. Conversely, positivity of cluster #4 for WFCD category is mainly determined by the high expression of *Wfdc21* gene that was shown to be among the top upregulated transcripts in vWAT [23]. Cluster #5, instead, contains cells exhibiting a strong transcriptional signature ascribable to the osteogenic compartment, including *Bglap*, *Spp1*, *Col1a1*, *Sparc* and *Alpl* (Table 2). Remarkably, such cell subpopulation was almost absent in the hypomorphic BM, which is consistent with the strongly reduced ability of mutant MSCs to generate osteoblasts in vitro. It is interesting to note that cells present within this cluster are positive also for CD200, a recently reported biomarker for periosteal stem cells, which are responsible for bone regeneration [24]. In addition, it is noteworthy that a recent paper [25], published after our initial submission, in which a different algorithm was utilized to calculate cluster abundance, showed that: i) ~12% of the clusters were of osteogenic nature, well corresponding to the 12.5% of our data and ii) osteogenic clusters contain 3.3% and 3.9% of the entire stroma dataset, respectively, similarly to our 4.9%.

Cluster #5, mostly unique to wt BM, is dependent on *Prep1*, since it virtually disappears when *Prep1* is downregulated in the BM milieu. This argues for Prep1 as having essential roles for cluster #5 survival. Its cells may either arise from progenitors that do normally express *Prep1,* or perhaps their survival may require Prep1-dependent mediators that are depleted in hypomorphic scenarios.

In addition, our analysis highlighted that cluster #7, although represented by few cells, basically includes cells that co-express *PDGFRα;* and *Sca1*, which trace mesenchymal stem/early progenitor cells [19,26]. Presence of *PDGFRα*- and *Sca1*-positive cells is not affected by the *Prep1^i/i^* genotype.

Cluster 2 also exhibits some adipogenic features, but to a much lower extent. Analysis of *Prep1* gene expression in wt BM shows that it is mostly expressed in cluster #2 and sporadically in clusters #1 and #5 (Supplementary Figure S1). *Prep1* downregulation does not majorly affect cluster #2 size and composition, however it affects overall cluster distribution: i) osteogenic cluster #5 is virtually missing; ii) cluster #4 is instead greatly expanded; and the new cluster #8 is detectable only in the hypomorphic BM (Figure 5B).

Overall, these data indicate that cluster #1 contains cells committed towards the fat cell lineage, whereas cluster 5 is essentially osteogenic. Moreover, in our view, clusters #2, #4 and #8, which partially express adipogenic genes, may represent earlier stages of differentiation.

Further studies, using single cell transcriptomics and functional validation assays are needed to shed light on mesenchymal progenitors gene signatures. Nevertheless, these initial scRNAseq analyses, mainly focused on identifying subpopulations belonging to the adipogenic and osteogenic lineages, are consistent and support our in vitro and in vivo observations. In addition, they represent the first description of single cell transcriptomics applied to study mesenchymal cell compartments in a mutant mouse model, as compared to its normal counterpart.

Taken together, our findings indicate that *Prep1* downregulation in the BM induces an enhanced adipogenesis and a concomitant reduced osteogenesis, and raise the hypothesis that Prep1 may be involved in the differentiation of mesenchymal stromal stem/progenitor cells, particularly controlling the adipo-osteogenic switch. This is in line with the widely accepted view that in the bone marrow adipogenesis and osteogenesis are alternative fates for mesenchymal stem/progenitor cells, and that transcription factors promoting one differentiation program concomitantly inhibit the alternative one [27].

Osteoblasts and adipocytes are essential components of the hematopoietic niche, thus, an altered adipo-osteo cell ratio may have implications also in the hematopoietic tissue, and at least partially account for the hematopoietic phenotypes detected in the adult *Prep1* mutants.

Since *Prep1^i/i^* BM cells appear to be enriched in cells of the adipogenic categories, we addressed the question of whether diminished *Prep1* expression could affect also adipose cells of other fat depots. Our histological analyses show that adipocytes of both subcutaneous and visceral WAT are smaller, storing fewer lipids in hypomorphic compared to control mice. In line with this, micro-CT images show significant, i.e., 39% whole-body fat volume depletion, in hypomorphic mice. These results are consistent with recent studies on heterozygous *Prep1^i/+^* reporting a 23% reduction in total body lipid content, and a higher percentage (30%) of small adipocytes in epididymal WAT, as compared to wt, resulting from the overexpression of adipogenic genes and leading to greater insulin sensitivity [28]. Notably, the proadipogenic transcriptomic profile observed in our hypomorphic BM is in line with these observations in hypomorphic WAT. We further extended fat characterization, by addressing BAT (beyond WAT) adiposity. We show that interscapular BAT has 30% greater density in hypomorphic than control mice. CT-derived tissue density is inversely proportional to the content of triglycerides, representing a recognized indicator of fat whitening/browning [17]. Thus, higher CT-density in hypomorphic BAT indicates a lower content of lipids, and greater degree of browning, which is in line with the smaller cell-size observed histologically. In turn, the degree of browning is a positive predictor of metabolic health [29]. In addition, our high-resolution transcriptomics analyses indicate that the total amount of hypomorphic cells (clusters 1, 2, 4 and 8) positive for BFCD genes is enriched by 4.5-folds, as compared to their wt counterparts. Overall, our findings in WAT and BAT are in agreement with, and contribute to explain previous evidence showing a multi-organ metabolic impact of Prep1, with e.g., higher insulin sensitivity in adipose cells, muscle and liver in *Prep1* mutants, offering protection against type 2 diabetes [29,30].

In conclusion, our results show that absence of physiological levels of *Prep1* is associated with alterations in mesenchymal BM cells, which are enriched in the adipogenic compartment, and defective in the osteogenic component. Therefore, Prep1 emerges as a potential balancing factor in mesenchymal cell fate choices. Furthermore, our data suggest that *Prep1* deficiency favours a pro-adipogenic phenotype and induces a “browning” effect in all fat tissues.

## 4. Materials and Methods

### 4.1. Histological Analysis of Bone Marrow and Adipose Tissues

#### 4.1.1. Bone Marrow

Femurs were collected in four mice (two hypomorphic, two control mice). They were fixed in 10% formalin for 48 h and then in 70% ethanol for 30 minutes. Samples were rinsed in distilled water and incubated with decalcifying solution for two hours (DiaPath S.p.A., Microdec, EDTA-Based, Ref. D0053, Martinengo, Bergamo, Italy). The decalcification process was ended when the bone was easily penetrated by a needle. Then, femoral bone marrow was processed and included in paraffin using the Donatello Diapath automatic tissue processor (Martinengo, Bergamo, Italy), sliced (HistoCore Autocut, Leica BioSystems microtome) with thickness of 2 μm, and stained with hematoxylin and eosin using the automated Dako CoverStainer (Santa Clara, CA, United States). Each section was documented at 10×, 20× and 40× magnification, by using the Olympus BX51 microscope and connected with an Olympus DP70 digital camera and AnalySIS 5.0 imaging system software (Olympus, Tokyo, Japan).

#### 4.1.2. Adipose Tissues

Three different adipose tissues were analysed in this study in three animals per group: interscapular brown adipose tissue (BAT), subcutaneous white adipose tissue (sWAT) and visceral adipose tissue (vWAT). Samples were dissected, fixed in 10% formalin for 24 h, dehydrated, embedded in paraffin (Bio-Optica, Milano, Italy), sliced (Microm HM 330) with 5 μm thickness, and stained with haematoxylin and eosin, according to standard protocols. Each section was documented at ×40 magnification using an Axioskop optical microscope connected with an AxioCam MRc5 colour-camera and AxioVision analysis software (Carl Zeiss, Oberkochen, Germany).

### 4.2. In Vitro Cultures of Mesenchymal Stromal Cells (MSCs)

Mice were sacrificed by cervical dislocation under general anaesthesia before collecting tibias and femurs. Bones were crushed, bone chips were digested with 1mg/mL Collagenase/Dispase (Roche 10269638001) for 45 minutes at 37 °C shaking. Bone marrow cells, cells isolated from bone chips digestion and bone chips were put in culture, using MesenCult Basal Medium supplemented with 20% Mesenchymal Mouse Stimulatory Supplement (Stem Cell Technologies) and 1% Pen-Strept (Life Technologies) (Complete Medium). Cells were grown at 37 °C in humidified atmosphere at 5% CO_2_. Medium was changed every three days and cells were trypsinized at confluence and reseeded at 2 × 10^4^ cells/cm^2^ (passage 1, p1). All experiments were performed at passage 2 of culture (p2). All the experimental protocols on mice were conducted in compliance with DL 26/2014: implementation of European Directive 2010/63 on the protection of animals used for scientific purposes. Animal studies were conducted in compliance with legislative decree 26/2014: implementation of the European directive 2010/63/EU on the protection of animals used for scientitic purposes. The study protocol was notified to, and approved by the Italian Ministry of Health in 2017 (number 65E5B.N.VPH).

### 4.3. Osteogenic Differentiation

Cells were seeded at p2 at 2 × 10^4^ cells/cm^2^ and at 90% confluence the Complete Medium was replaced with osteogenic induction medium (MesenCult basal medium supplemented with 20% osteogenic stimulatory supplement (StemCell Technologies, Vancouver, Canada) and 1% Pen-Strept (Life Technologies, Carlsbad, CA, USA)). Osteogenic differentiation efficiency was evaluated by Alizarin Red S’ Staining.

### 4.4. Single Cell RNA Sequencing and Bioinformatics Analysis

MSCs were depleted for CD45, Ter119 by using a magnetic MACS Separator (LS Columns, Miltenyi Biotec 130-042-401). Cells were first labelled with Biotin anti-mouse CD45.2 (Biolegend 109803), Biotin anti-mouse Ter119 (Biolegend 116204) for 20 minutes on ice, then incubated with anti-biotin beads (Biolegend 130-090-485) for 20 minutes on ice. Cell suspension was applied onto the column. Unlabelled cells corresponding to the CD45 and Ter119 negative fraction was collected. After the depletion, CD45, Ter119 and CD31 negative cells were FACS-purified (CD45.2-APC Biolegend 109814, Ter-119-APC Biolegend 116212, CD31-APC Biolegend 102410). Live cells were gated using DAPI staining.

FACS-purified cells were used to perform Single Cell RNA sequencing using 10X Genomics platform. Five hundred cells from each mouse were loaded into one channel of the Chromium system, and libraries prepared according to manufacturer’s protocol (10X Genomics). Illumina sequencing was performed by Novaseq (350,000 reads per cells). Cell Ranger version 1.3 (10X Genomics, Pleasanton, California, United States) was utilized to process raw sequencing data. Sequence reads from the two mice were pooled to build a unique database which amounted to a total of 363,478 reads from a total of 454 cells, with a median number of 2017 genes per cell. Loupe Cell Browser (Pleasanton, California, United States) was used in order to visualize further downstream analyses. Resulting figures depict level of expression for each signature/category calculated as ≥50% of Log2 Feature Max as visualized on Loupe Cell browser.

### 4.5. Whole-Body Micro-CT Imaging and Analysis

The body composition of all mice in terms of bone tissue, fat mass and lean mass was measured by X-ray micro-computed tomography (micro-CT). In order to reduce motion artifacts due to respiration and cardiac motion, all whole-body scans were performed right after the animal sacrifice and before harvesting the tissue components for subsequent ex vivo and in vitro analysis. The IRIS-CT scanner (Inviscan SAS, Strasbourg, France) was used for this purpose. The following scan parameters were used: 65 kV, 1 mA, 1280 views over 360°, for a total scan time of 90 s per mouse. Volumetric images were reconstructed using cone-beam filtered backprojection (FBP) with standard ramp filter, using corrections for beam hardening and ring artifacts. Reconstructions were done on a field of measurement (FOM) of 47 × 47 × 114 mm and with an isotropic voxel size of 58.8 micron. Image segmentation was performed using the software Seg3D v 2.4. The following tissue components were segmented: bone, lung and airways, total adipose tissue, interscapular brown adipose tissue (BAT). Only slices comprised between the first cervical vertebra and the first caudal vertebra were considered for the segmentation. For each segmented tissue, the radiodensity was calculated as the average CT number expressed in Hounsfield Units (HU). The radiodensity was used as an indirect metric of tissue mass density due its good linear relationship in the density range of soft tissues [31]. The total mouse length was also measured from the tip of the nose to the first caudal vertebra, using a multi-segment broken line tool in ImageJ [32] that allowed tracking the entire vertebral column along its specific curvature. Mann–Whitney nonparametric test between the two groups of mice was performed for each measured parameter. The threshold for statistical significance was set to *p* = 0.05. All statistical analyses were performed in R ver. 3.5.3.

## Figures and Tables

**Table 1:** Summary of the in vivo morphometric analysis. Comparison between the two groups for each variable are performed by Mann-Whitney non-parametric test. An asterisk (*) after the *p*-value denotes a significant difference between groups.

	WT (*n* = 16)	Prep1^*i/i*^ (*n* = 14)	*p*-Value
**Weight (g)**			
mean (sd)	32.232 ± 5.759	27.479 ± 4.022	0.029 (*)
min	19.89	21.24	
max	40.21	32.78	
**Total volume (cm^3^)**			
mean (sd)	31.421 ± 5.794	26.496 ± 4.132	0.026 (*)
min	19.261	20.065	
max	39.37	32.23	
**Total fat volume (cm^3^)**			
mean (sd)	7.641 ± 3.487	4.655 ± 2.367	0.029 (*)
min	1.948	1.281	
max	14.330	7.724	
**Total bone volume (cm^3^)**			
mean (sd)	1.411 ± 0.164	1.386 ± 0.169	1
min	1.155	1.112	
max	1.728	1.644	
**Length (mm)**			
mean (sd)	96.511 ± 2.763	94.561 ± 2.505	0.044 (*)
min	90.26	90.17	
max	101.80	98.54	
**% Fat**			
mean (sd)	21.724 ± 7.614	15.540 ± 6.843	0.021 (*)
min	6.74	5.72	
max	35.13	27.42	
**BAT volume (cm^3^)**			
mean (sd)	0.106 ± 0.055	0.088 ± 0.042	0.57
min	0.043	0.045	
max	0.229	0.208	
**Bone radiodensity (HU)**			
mean (sd)	1461 ± 113	1400 ± 165	0.22
min	1244	1204	
max	1661	1661	
**Fat radiodensity (HU)**			
mean (sd)	−260 ± 29	−248 ± 25	0.41
min	−335	−278	
max	−214	−196	
**BAT radiodensity (HU)**			
mean (sd)	−180 ± 46	−132 ± 36	0.013 (*)
min	−236	−182	
max	−91	−54	

**Table 2:** Gene signatures characterizing the indicated GO categories, or the LEPR+ Adipo cells.

GO WFCD	LEPR+ Adipo	GO BFCD		Progenitors	Osteo
Cebpα	Hp	Adipoq	Mecom	PDGFRα	Cd200
Ctbp1	Lpl	Adrb1	Metrnl	Ly6a	Col1a1
Ctbp2	Adipoq	Adrb2	Mrap		Col1a2
Fabp4	Slc1a5	Adrb3	Mtor		Alpl
Fgf10	Cd302	Aldh6a1	Napepld		Spp1
Ncor2	Gas6	Arl4a	Nudt7		Sparc
Per2	Apoe	Bnip3	Pex11a		Msx2
Pparγ	Lepr	Cebpα	Plac8		Bglap
Prdm16		Cebpβ	Pparγ		Hox10
Scd1		Dusp10	Pparγc1a		Sp7
Sirt1		Ebf2	Prdm16		Runx2
Snai2		Ero1l	Ptgs2		
Tbl1xr1		Fabp4	Rarres2		
Wfdc21		Fndc5	Rgs2		
Cebpδ		Fto	Scd1		
Cebpβ		Hnrnpu	Selenbp1		
Adipoq		Itga6	Sh2b2		
Srebf1		Lama4	Sirt1		
		Lamb3	Slc2a4		
		Lep	Trpv4		
		Lrg1	Vstm2a		
		Mapk14	Zbtb7b		
		Mb	Zfp516		

GO, Gene Ontology; WFCD, White Fat Cell Differentiation; LEPR, Leptin Receptor; BFCD, Brown Fat Cell Differentiation.

## Supplementary Materials

Supplementary materials can be found at https://www.mdpi.com/1422-0067/20/15/3639/s1.
