# Supplementary material for: The Role of Prep1 in the Regulation of Mesenchymal Stromal Cells
Source: Int J Mol Sci. 2019 Jul 25;20(15):3639. doi: 10.3390/ijms20153639 (PMC6696203; doi:10.3390/ijms20153639)
Supplement: Supplementary file 1 [file ijms-20-03639-s001.pdf]

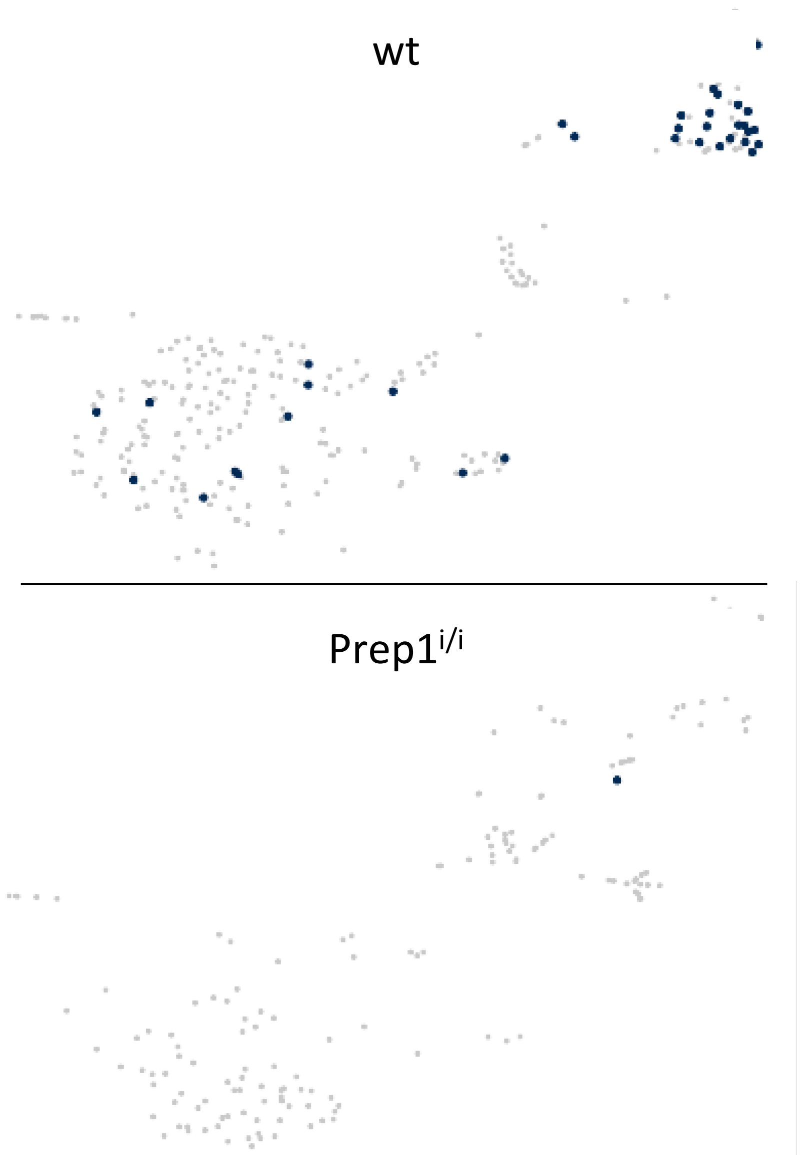

Supplementaty Figure S1. Prep1 expression distribution. t-SNE plot shows that Prep1 is mainly expressed in wt cluster 2 and, as expected, it is almost undetectable in the hypomorphic sample.
